# Does the SORG Machine-learning Algorithm for Extremity Metastases Generalize to a Contemporary Cohort of Patients? Temporal Validation From 2016 to 2020

**DOI:** 10.1097/CORR.0000000000002698

**Published:** 2023-05-25

**Authors:** Tom M. de Groot, Duncan Ramsey, Olivier Q. Groot, Mitchell Fourman, Aditya V. Karhade, Peter K. Twining, Emily A. Berner, Brian P. Fenn, Austin Keith Collins, Kevin Raskin, Santiago Lozano, Eric Newman, Marco Ferrone, Job N. Doornberg, Joseph H. Schwab

**Affiliations:** 1 Massachusetts General Hospital, Boston, MA, USA; 2 University Medical Center Groningen, Groningen, the Netherlands; 3 University of Texas RGV School of Medicine, Edinburg, TX, USA; 4 Brigham and Women’s Hospital, Boston, MA, USA

## Abstract

**Background:**

The ability to predict survival accurately in patients with osseous metastatic disease of the extremities is vital for patient counseling and guiding surgical intervention. We, the Skeletal Oncology Research Group (SORG), previously developed a machine-learning algorithm (MLA) based on data from 1999 to 2016 to predict 90-day and 1-year survival of surgically treated patients with extremity bone metastasis. As treatment regimens for oncology patients continue to evolve, this SORG MLA-driven probability calculator requires temporal reassessment of its accuracy.

**Question/purpose:**

Does the SORG-MLA accurately predict 90-day and 1-year survival in patients who receive surgical treatment for a metastatic long-bone lesion in a more recent cohort of patients treated between 2016 and 2020?

**Methods:**

Between 2017 and 2021, we identified 674 patients 18 years and older through the ICD codes for secondary malignant neoplasm of bone and bone marrow and CPT codes for completed pathologic fractures or prophylactic treatment of an impending fracture. We excluded 40% (268 of 674) of patients, including 18% (118) who did not receive surgery; 11% (72) who had metastases in places other than the long bones of the extremities; 3% (23) who received treatment other than intramedullary nailing, endoprosthetic reconstruction, or dynamic hip screw; 3% (23) who underwent revision surgery, 3% (17) in whom there was no tumor, and 2% (15) who were lost to follow-up within 1 year. Temporal validation was performed using data on 406 patients treated surgically for bony metastatic disease of the extremities from 2016 to 2020 at the same two institutions where the MLA was developed. Variables used to predict survival in the SORG algorithm included perioperative laboratory values, tumor characteristics, and general demographics. To assess the models’ discrimination, we computed the c-statistic, commonly referred to as the area under the receiver operating characteristic (AUC) curve for binary classification. This value ranged from 0.5 (representing chance-level performance) to 1.0 (indicating excellent discrimination) Generally, an AUC of 0.75 is considered high enough for use in clinical practice. To evaluate the agreement between predicted and observed outcomes, a calibration plot was used, and the calibration slope and intercept were calculated. Perfect calibration would result in a slope of 1 and intercept of 0. For overall performance, the Brier score and null-model Brier score were determined. The Brier score can range from 0 (representing perfect prediction) to 1 (indicating the poorest prediction). Proper interpretation of the Brier score necessitates a comparison with the null-model Brier score, which represents the score for an algorithm that predicts a probability equal to the population prevalence of the outcome for each patient. Finally, a decision curve analysis was conducted to compare the potential net benefit of the algorithm with other decision-support methods, such as treating all or none of the patients. Overall, 90-day and 1-year mortality were lower in the temporal validation cohort than in the development cohort (90 day: 23% versus 28%; p < 0.001, and 1 year: 51% versus 59%; p<0.001).

**Results:**

Overall survival of the patients in the validation cohort improved from 28% mortality at the 90-day timepoint in the cohort on which the model was trained to 23%, and 59% mortality at the 1-year timepoint to 51%. The AUC was 0.78 (95% CI 0.72 to 0.82) for 90-day survival and 0.75 (95% CI 0.70 to 0.79) for 1-year survival, indicating the model could distinguish the two outcomes reasonably. For the 90-day model, the calibration slope was 0.71 (95% CI 0.53 to 0.89), and the intercept was -0.66 (95% CI -0.94 to -0.39), suggesting the predicted risks were overly extreme, and that in general, the risk of the observed outcome was overestimated. For the 1-year model, the calibration slope was 0.73 (95% CI 0.56 to 0.91) and the intercept was -0.67 (95% CI -0.90 to -0.43). With respect to overall performance, the model’s Brier scores for the 90-day and 1-year models were 0.16 and 0.22. These scores were higher than the Brier scores of internal validation of the development study (0.13 and 0.14) models, indicating the models’ performance has declined over time.

**Conclusion:**

The SORG MLA to predict survival after surgical treatment of extremity metastatic disease showed decreased performance on temporal validation. Moreover, in patients undergoing innovative immunotherapy, the possibility of mortality risk was overestimated in varying severity. Clinicians should be aware of this overestimation and discount the prediction of the SORG MLA according to their own experience with this patient population. Generally, these results show that temporal reassessment of these MLA-driven probability calculators is of paramount importance because the predictive performance may decline over time as treatment regimens evolve. The SORG-MLA is available as a freely accessible internet application at https://sorg-apps.shinyapps.io/extremitymetssurvival/.

*Level of Evidence* Level III, prognostic study.

## Introduction

Metastatic bone disease affects between 280,000 and 400,000 people each year in the United States [7, 8, 14, 30]. Metastatic bone disease is a major source of morbidity and mortality and is a growing economic burden; its cost was estimated to be USD 12.6 billion in 2007, or roughly 17% of all direct medical costs in cancer care [20]. Survival after the diagnosis of a bone metastasis may be measured in months, or it may be measured in years. Knowing which patients are likely to survive for long periods after this diagnosis is important to help them make personal plans and determine care decisions, such as whether to preemptively stabilize impending pathologic fractures. However, patients with an estimated survival of less than 3 months may have a relative contraindication to prophylactic stabilization because morbidity, the short-term complications of stabilization, and costs must be weighed against that of fracture risk during this shorter time span [16]. Those expected to live more than 1 year must contend with the risks of longer-term complications such as nonunion or implant failure as well as short-term complications and morbidity, which could heavily influence the choice of implant or the choice between temporal stabilization or arthroplasty [10].

Several attempts have been made to create predictive models for survival in these patients. Methods have included classic statistics [27] and more complex ones such as boosting algorithms [11] and Bayesian belief networks [4]. Machine learning has also been applied in order to answer this question [24]. Machine learning is an area of artificial intelligence research that can be used to develop predictive models based on large datasets. These methods have been used extensively in areas from diagnostic radiology to orthopaedic surgery [1, 5, 17, 18, 26]. We, the Skeletal Oncology Research Group (SORG), previously developed a machine-learning algorithm (MLA) to predict 90-day and 1-year survival of surgically treated patients with extremity bone metastasis [24]. This was based on 1090 patients treated at two tertiary institutions between 1999 and 2016. The 15 variables used in this model were age, primary tumor, visceral or brain metastases, use of systemic therapy, and 10 laboratory values including albumin and lymphocyte count. However, prediction models based on these historical cohorts are at risk of becoming outdated, because there have been major advancements in cancer biology and treatment over the past decade. It is therefore important to assess how these models perform in patients from more recent time periods to determine whether these models are still relevant. Although the SORG MLA has been validated internationally [22, 29], it has not been tested on a contemporary cohort.

We therefore sought to evaluate the model’s performance on a newer cohort of patients who were surgically treated for extremity metastatic disease of bone between 2016 and 2020. In this study, we asked: Does the SORG-MLA accurately predict 90-day and 1-year survival in patients who receive surgical treatment for a metastatic long-bone lesion in a more recent cohort of patients treated between 2016 and 2020?

## Patients and Methods

### Study Design and Setting

This retrospective, comparative study was performed at a high-volume, urban tertiary care center in the northeastern United States (Massachusetts General Hospital and Brigham and Women’s Hospital, both located in Boston, MA, USA) in accordance with the Transparent Reporting of a Multivariable Prediction Model for Individual Prognosis or Diagnosis [15] and the Strengthening the Reporting of Observational Studies in Epidemiology [2] guidelines.

### Patients

**Fig. 1:**
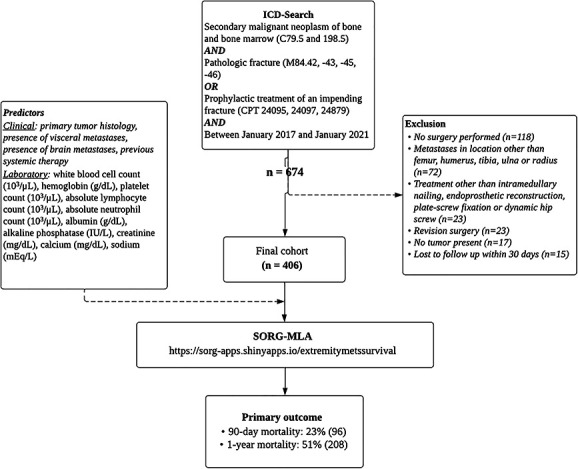
This flowchart shows the patients included in this study.

Between 2017 and 2021, we identified 674 patients who were 18 years and older through the ICD codes for secondary malignant neoplasm of bone and bone marrow and the CPT codes for completed pathologic fractures or prophylactic treatment of an impending fracture (Fig. 1). Exclusion criteria were no surgery (n = 118); metastases in a location other than the femur, humerus, tibia, ulna, or radius (n = 72); treatment other than intramedullary nailing, endoprosthetic reconstruction, plate-screw fixation, or dynamic hip screw (n = 23); revision surgery (n = 23); no tumor present (n = 17); and loss to follow-up within 1 year (n = 15). Completed pathologic fractures and impending pathologic fractures were included. A multidisciplinary team of medical oncologists, anesthesiologists, and orthopaedic surgeons were responsible for assessing patients’ ability to undergo surgery. An impending pathologic fracture was assessed by an orthopaedic surgeon and radiologist with the use of clinical and radiographic features of the lesion. Applying the inclusion and exclusion criteria resulted in 406 patients who underwent surgical treatment for a long-bone metastasis (Fig. 1).

### Baseline Characteristics

The median patient age in the cohort was 67 years (IQR 59 to 74 years), and 54% (221 of 406) of patients were female (Table 1). Regarding primary tumor histology, 34% (137 of 406) of the patients were in the slow-growth group, 32% (131 of 406) were in the moderate-growth group, and 34% (138 of 406) were in the rapid-growth group. Most lesions were in the lower extremity (79% [321 of 406]). Most patients were treated with an intramedullary nail (58% [237 of 406]), 28% (114 of 406) of the patients received endoprosthetic reconstruction, 8% (31 of 406) of the patients were treated with plate-screw fixation, 5% (21 of 406) of the patients had multiple implants, and 1% (3 of 406) were treated with a dynamic hip screw.

**Table 1.** Baseline comparison between temporal validation (n = 406) and development population (n = 1090)

Variable	Temporal validation (n = 406)	Development (n = 1090)	p value
Age in years	67 (59-74)	63 (54-72)	< 0.001
Female sex	54 (221)	56 (610)	0.63
Primary tumor type^a^			0.002
Slow growth	34 (137)	42 (460)	
Moderate growth	32 (131)	24 (263)	
Rapid growth	34 (138)	34 (367)	
Location			0.31
Lower extremity	79 (321)	77 (835)	
Upper extremity	21 (85)	23 (255)	
Surgical treatment			< 0.001
Intramedullary nail	59 (237)	58 (636)	
Endoprosthetic reconstruction	28 (114)	22 (241)	
Plate-screw fixation	8 (31)	14 (155)	
Dynamic hip screw	1 (3)	2 (21)	
Multiple implants	5 (21)	3 (37)	
Visceral metastases	45 (182)	45 (487)	0.97
Brain metastases	16 (63)	16 (175)	0.80
Pathologic fracture	48 (194)	55 (595)	0.02
Previous systemic therapy	67 (270)	62 (676)	0.11
Preoperative laboratory values^b^			
Albumin level (g/dL)	3.7 (3.3-4.0)	3.7 (3.2-4.1)	0.43
Alkaline phosphatase level (IU/L)	106 (81-157)	101 (74-146)	0.047
Calcium (mg/dL)	9.3 (8.9-9.7)	9.2 (8.7-9.7)	0.002
Creatinine (mg/dL)	0.8 (0.7-1.0)	0.8 (0.7-1.1)	0.11
Hemoglobin level (g/dL)	11.2 (9.8-12.7)	11.3 (10.0-12.6)	0.66
Lymphocyte absolute count (10^3^/uL)^c^	1.0 (0.6-1.5)	1.0 (0.6-1.5)	0.19
Neutrophil absolute count (10^3^/uL)	5.3 (3.7-7.5)	5.5 (3.7-7.8)	0.62
Platelet count (10^3^/uL)	246 (180-324)	251 (184-332)	0.41
Sodium (mg/dL)	138 (136-140)	138 (136-140)	0.02
White blood cell count (10^3^/uL)	7.4 (5.5-10.1)	7.3 (5.2-9.9)	0.53
Mortality^b^			
90-day	23 (92)	28 (305)	< 0.001
1-year	51 (208)	59 (643)	< 0.001

Data presented as % (n) or median (IQR). Baseline characteristics were compared using the chi-square test for categorical variables, the Mann-Whitney U test for continuous variables, and Cox regression for 90-day and 1-year mortality.

a Slow growth includes hormone-dependent breast cancer, hormone-dependent prostate cancer, malignant lymphoma, malignant myeloma, and thyroid cancer; moderate growth includes nonsmall cell lung cancer with molecularly targeted therapy, hormone-independent breast cancer, hormone-independent prostate cancer, renal cell carcinoma, sarcoma, other gynecologic cancer, and others; and rapid growth includes other lung cancer, colon and rectal cancer, gastric cancer, hepatocellular carcinoma, pancreatic cancer, head and neck cancer, other urologic cancer, esophageal cancer, malignant melanoma, gallbladder cancer, cervical cancer, and unknown origin.

b Missing data in temporal validation cohort: albumin 17% (70 of 406), alkaline phosphatase 17% (70 of 406), calcium 4% (17 of 406), creatinine 4% (17 of 406), hemoglobin 3% (12 of 406), lymphocyte count 14% (56 of 406), neutrophil count 14% (56 of 406), platelet count 3% (13 of 406), sodium 4% (16 of 406), white blood cell count 3% (12 of 406), and vital status at 90 days 1% (four of 406) and 1 year 4% (15 of 406).

Forty-five percent (182 of 406) of patients had visceral metastases and 16% (63 of 406) had brain metastases. Twenty-three percent (92 of 406) died within 90 days and 51% (208 of 406) died within 1 year. Patients in the validation cohort were older than those in the development cohort (median age 67 years [IQR 59 to 74] versus 63 years [IQR 54 to 72]) (Table 1). Patients in the validation cohort also presented with slow-growth tumors less often than those in the development cohort (34% [137 of 406] versus 42% [460 of 1090]) and more often with moderate growth tumor types (32% [131 of 406] versus 24% [263 of 1090]). Additionally, patients in the validation cohort presented with a pathologic fracture less often than those in the development cohort (48% [194 of 406] versus 55% [595 of 1090]).

### Outcome and Explanatory Variables

As in the developmental cohort of the SORG-MLA, the primary outcomes were 90-day and 1-year mortality. Mortality was defined as the time between surgical treatment for a long-bone metastasis and death of any cause. The date of death or date of the last follow-up was retrieved from electronic healthcare records and the Social Security Death Index.

We manually obtained the 15 required input variables used in the SORG-MLA by reviewing operative notes, medical records, radiology reports, and pathology: sex, age, histologic subtype of the primary tumor (classified into the following three groups: slow growth, moderate growth, and rapid growth as classified by Katagiri et al. [13]), tumor location, visceral metastases, brain metastases, previous systemic therapy, and 12 preoperative laboratory values. Systemic therapy was defined as having received at least one of the following: chemotherapy, targeted therapy, hormone therapy, or immunotherapy. Preoperative laboratory values comprised absolute lymphocyte count (x10^3^/uL), absolute neutrophil count (x10^3^/uL), albumin level (g/dL), alkaline phosphatase (IU/L), calcium (mg/dL), creatinine (mg/dL), hemoglobin level (g/dL), neutrophil-to-lymphocyte ratio, platelet count (x10^3^/uL platelet-to-lymphocyte count, sodium (mg/dL) levels, and white blood cell count (x10^3/^uL).

### Ethical Approval

Ethical approval for this study was obtained from the institutional review board at Massachusetts General Hospital, Boston, MA, USA (2018P000688).

### Statistical Analysis

We assessed differences in baseline characteristics between the development cohort [24] and the temporal validation cohort (patients from the same institution with the same inclusion and exclusion criteria, except the temporal validation cohort included patients from a more recent period) using the chi-square test for categorical variables and the Mann-Whitney U test for continuous variables.

After running the algorithm’s code on the dataset, we analyzed and compared the predictions of all individual patients with actual 90-day and 1-year mortality rates. Algorithm performance was assessed by the same metrics used in the developmental study and as recommended by the Transparent Reporting of a Multivariable Prediction Model for Individual Prognosis or Diagnosis guidelines [15]: discrimination using the area under the curve (AUC); calibration by using a visual plot, intercept, and slope; overall performance using a Brier score and a null model Brier score; and a decision curve analysis.

The AUC, which is a measure of the ability of the model to distinguish (discriminate) between positive and negative outcomes (in this study, the occurrence of death is the positive outcome) was visualized by plotting receiver operating characteristic curves. The AUC ranges from 0.5 (no better than a coin toss) to 1.0 (perfect discrimination). In general, an AUC of 0.9 to 1.0 represents excellent discrimination, 0.8 to 0.9 good, 0.7 to 0.8 reasonable, 0.6 to 0.7 fair, and 0.5 to 0.6 poor, where 0.75 is generally accepted as a prediction model that can aid physicians in their predictions [3]. Calibration of a prediction model represents the agreement between the predicted versus the actual outcome (in this case, 90-day and 1-year mortality). This was visualized by plotting the predicted probabilities (x-axis) against the observed outcome (y-axis). The calibration curve provides an intercept and a slope. The calibration intercept indicates the extent to which predictions are systematically too low or too high. A negative intercept indicates overestimation of the observed outcome. The calibration slope indicates agreement between the predicted probability and observed outcome. In a model that is calibrated perfectly, the slope equals 1. However, if the calibration slope is less than 1, it suggests that the predicted risks were overly extreme, overestimating for high-risk patients and underestimating for low-risk patients.

The Brier score measures the overall performance of the algorithm by calculating the mean squared difference between predicted probabilities and actual outcomes. A lower score indicates more accurate predictions, while a high score suggests the predicted probabilities are unreliable. In a binary classification, a perfect model would have a Brier score of 0 and a completely random model would have a score of 0.25. When evaluating the Brier score, the prevalence of outcome must also be considered, which is why a null-model Brier score was calculated by assigning a probability equal to the prevalence of the outcome to each patient to serve as a reference point. Decision curve analysis provided a framework to judge the relative value of benefits and harms associated with the prediction model. It aims to determine whether the benefits of using a model outweigh the costs of either treating all or no patients. Decision curve analysis charts the net benefit of a model across a range of threshold probabilities that represent the minimum likelihood of an event that would warrant a specific treatment or intervention. By doing so, decision curve analysis provides useful insights into the clinical impact of using a model to guide decision-making, helping healthcare providers decide whether to adopt a model in their practice.

Lastly, an F1 score was calculated for both models. The F1 score is a performance metric commonly used in evaluating classification models. It considers precision and recall and reflects how well the model can correctly identify positive cases while minimizing the number of false positives. A higher F1 score generally indicates a more accurate and reliable model, while a lower score suggests that the model may need improvement.

The missForest method [13] was used to impute missing values for the following variables: albumin (17% [70 of 406]), alkaline phosphatase (17% [70 of 406]), calcium (4% [17 of 406]), creatinine (4% [17 of 406]), hemoglobin (3% [12 of 406]), lymphocyte count (14% [56 of 406]), neutrophil count (14% [56 of 406]), platelet count (3% [13 of 406]), sodium (4% [16 of 406]), and white blood cell count (3% [12 of 406]). Vital status at 90 days was missing in 1% (4 of 406) and in 4% (15 of 406) at 1 year.

All statistical analyses were performed using Python (Python Software Foundation). A two‐tailed p value less than 0.05 was considered significant.

## Results

Four-hundred six patients were included in the final analysis. The median age was 67 years (IQR 59 to 74 years) and 54% (221 of 406) of the patients were female. Forty-eight percent (194) of the patients presented with a pathologic fracture, and the mortality percentage was 23% (92) for the 90-day timepoint and 51% (208) for the 1-year timepoint.

### Ability of the SORG-MLA to Predict 90-day and 1-year Survival in Temporal Validation

**Fig. 2:**
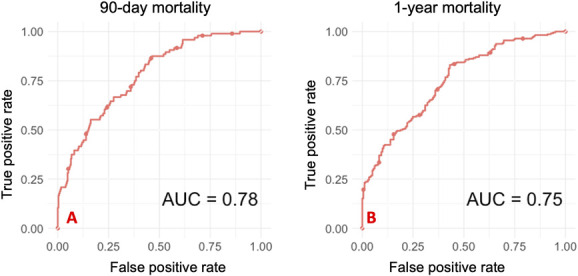
These graphs show discrimination of the SORG-MLA for extremity metastasis on temporal validation (n = 406). The area under the receiver operating characteristic curves (AUC) show decreased discriminative performance for (**A**) 90-day mortality and (**B**) 1-year mortality.

The SORG-MLA showed a decreased ability to accurately predict the postoperative 90-day and 1-year mortality. AUC, which is a measure of discrimination (a perfect model has an AUC of 1 and 0.5 represents a model that can discriminate no better than a coin toss) was 0.78 (95% CI 0.72 to 0.82) for 90-day mortality and 0.75 (95% CI 0.70 to 0.79) for 1-year mortality (Table 2). Generally speaking, a model with an AUC of 0.75 is considered suitable for clinical use. The calibration analysis provided an intercept of -0.66 (95% CI -0.94 to -0.39) (indicating overestimated predictions) and slope of 0.71 (95% CI 0.53 to 0.89) (suggesting overly extreme predictions) for 90-day mortality prediction. For 1-year mortality, calibration showed an intercept of -0.67 (95% CI -0.90 to -0.43) and a slope of 0.73 (95% CI 0.56 to 0.91) (Fig. 2). The actual 90-day and 1-year mortality rates in the temporal validation cohort were lower than the predicted values (90 days: 23% versus 28%; p < 0.001, and 1 year: 51% versus 59%; p < 0.001), which are represented by the negative calibration intercepts. Calibration assesses the agreement between predicted and actual outcomes, with a perfect model having a slope of 1.

**Table 2.** Performance of the SORG machine-learning algorithm for extremity metastasis on temporal validation (n = 406)

Metric	90-day mortality (95% CI)	1-year mortality (95% CI)
	Discrimination	
AUC^a^	0.78 (0.72 to 0.82)	0.75 (0.70 to 0.79)
F1 score^b^	0.52 (0.44 to 0.59)	0.76 (0.70 to 0.81)
	Calibration	
Intercept^c^	-0.66 (-0.94 to -0.39)	-0.67 (-0.90 to -0.43)
Slope^d^	0.71 (0.53 to 0.89)	0.73 (0.56 to 0.91)
	Overall performance	
Brier score^e^	0.16 (0.14 to 0.18)	0.22 (0.19 to 0.24)
Null-model Brier score	0.18	0.25

a When the AUC = 1, the discrimination is perfect; when the AUC = 0.5, the model predicts no better than a simple coin toss.

b F1 score: Combines the precision (true positives divided by all predicted positives) and recall (true positives divided by all actual positives), thereby computing how many times a model made a correct prediction across the entire dataset.

c Intercept: Indicates the extent to which predictions are systematically too low or too high. A negative intercept indicates overestimation of the outcome.

d Slope: Indicates agreement between the predicted probability and observed outcome. In a model that is calibrated perfectly, the slope equals 1. However, if the calibration slope is less than 1, it suggests the predicted risks were overly extreme, overestimating for high-risk patients and underestimating for low-risk patients.

e Brier score: Measures the overall performance of the algorithm. A lower score indicates more accurate predictions, while a high score suggests the predicted probabilities are unreliable. In binary classification, a perfect model would have a Brier score of 0, and a completely random model would have a score of 0.25. When evaluating the Brier score, the prevalence of an outcome must also be considered; that is why a null-model Brier score was calculated by assigning a probability equal to the prevalence of the outcome to each patient to serve as a reference point.

**Fig. 3:**
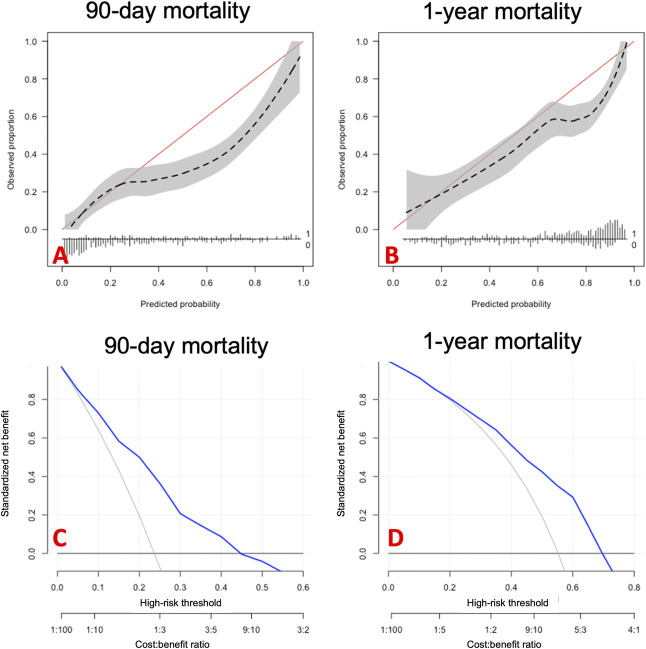
These graphs show the calibration and decision curve analysis of the SORG-MLA for extremity metastasis on temporal validation (n = 406). The calibration curves indicate overestimation of mortality for (**A**) 90 days in probabilities between 0.30 and 1.0 and (**B**) between 0.7 and 1.0 for 1-year mortality, indicated by the negative intercept. (**C and D**) The decision curve analysis showed a greater net benefit for all predicted probabilities compared with default strategies of changing management for all patients or no patients (blue line) compared with changing the treatment for all patients (slanted gray line) or for no patients (horizontal black line). A color image accompanies the online version of this article.

The Brier score measures the overall performance of the algorithm, with a lower score indicating more accurate predictions. The prevalence of the outcome should also be considered when evaluating the Brier score, and a null-model Brier score serves as this reference point. Ninety-day mortality prediction was 0.16 (95% CI 0.14 to 0.18) compared with a higher null-model Brier score of 0.18, indicating greater performance of the SORG-MLA. For 1-year mortality prediction, the Brier score was 0.22 (95% CI 0.19 to 0.24), which was also lower than the null-model Brier score of 0.25 (Table 2). The decision curve analysis showed greater net benefit at all predicted probabilities compared with default strategies of changing management for all patients or no patients. Above the threshold of 0.7, the predictions of the SORG-MLA resulted in a larger net benefit when predicting 1-year mortality (Fig. 3C and 3D).

### Overestimation of Mortality

The negative calibration intercept of the SORG-MLA suggests overestimation of mortality for 90-day and 1-year survival (Fig. 3). The SORG-MLA tended to overestimate 90-day and 1-year mortality in 9% (38 of 406) of patients (Table 3). Overestimation was in this regard defined as every false-positive prediction across our patient population. A subgroup analysis of these patients showed they were more often treated with novel treatment strategies such as PD-(L)1 inhibitors and cyclin-dependent kinase inhibitors (84% [32 of 38] in the overestimated group versus 55% [204 of 368] in the nonoverestimated group; p = 0.001). Moreover, the response of these patients to the novel treatment regimens was also better; 76% (29 of 38) of patients with overestimated mortality had a good response to the immunotherapy, compared with 23% (86 of 368) of the patients with a normal mortality prediction (p < 0.001) (Table 3).

**Table 3.** Comparison of patients with overestimated mortality and patients with acceptable prediction of mortality

**Variable**	**Adequate prediction (n = 368)**	**Overestimated mortality (n = 38)**	**p value**
Age in years	66 (60-72)	68 (61-73)	0.21
Female sex	55 (201)	53 (20)	0.94
Primary tumor type			0.29
Slow growth	34 (125)	32 (12)	
Moderate growth	33 (122)	24 (9)	
Rapid growth	33 (121)	45 (17)	
Treatment with immunotherapy	55 (204)	84 (32)	0.001
Response to immunotherapy	23 (86)	76 (29)	< 0.001
Primary tumor histology			
Breast	24 (89)	18 (7)	0.55
Hormone-dependent	21 (79)	16 (6)	0.54
Hormone-independent	4 (13)	3 (1)	0.99
Lung	23 (85)	26 (10)	0.81
Renal cell	13 (48)	8 (3)	0.51
Prostate	7 (27)	8 (3)	0.99
Hormone-dependent	4 (13)	5 (2)	0.93
Hormone-independent	4 (15)	3 (1)	0.99
Sarcoma	5 (18)	0	0.24
Multiple myeloma	4 (13)	3 (1)	0.99
Thyroid	3 (10)	0	0.61
Hepatocellular	2 (6)	3 (1)	0.99
Head or neck	2 (9)	3 (1)	0.99
Melanoma	2 (9)	5 (2)	0.56
Lymphoma	3 (10)	8 (3)	0.21
Other	2 (9)	3 (1)	0.99
Colic	2 (7)	3 (1)	0.99
Gynecologic	2 (7)	0	0.84
Bladder	1 (5)	3 (1)	0.51
Gallbladder	1 (4)	0	0.99
Esophageal	1 (3)	0	0.99
Gastric	1 (2)	0	0.99
Unknown origin	1 (3)	5 (2)	0.11
Pathologic fracture	47 (172)	58 (22	0.26
Tumor location			0.10
Femur	80 (293)	74 (28)	
Humerus	18 (66)	24 (9)	
Tibia	2 (9)	2 (1)	
Metastases			
Visceral metastases	45 (167)	37 (14)	0.18
Brain metastases	15 (54)	24 (9)	0.22
Previous systemic therapy	66 (242)	74 (28)	0.42
Laboratory values			
Absolute lymphocyte count (10^3^/uL)	0.97 (0.58-1.54)	0.78 (0.44-1.46)	0.11
Absolute neutrophil count (10^3^/uL)	5.4 (3.8-7.6)	5.8 (4.0-9.2)	0.40
Albumin level (g/dL)	3.7 (3.3-4.0)	3.4 (3.1-3.9)	0.06
Alkaline phosphatase level (IU/L)	105 (82-154)	141 (108-208)	< 0.01
Calcium (mg/dL)	9.3 (8.9-9.7)	9.0 (8.4-9.4)	< 0.001
Creatinine (mg/dL)	0.79 (0.65-1.00)	0.75 (0.66-0.91)	0.61
Hemoglobin level (g/dL)	11.2 (9.7-12.6)	10.3 (9.2-11.9)	< 0.05
Neutrophil-to-lymphocyte ratio	5.8 (3.1-9.9)	7.0 (3.8-13.3)	0.17
Platelet count (10^3^/uL)	250 (185-324)	244 (170-354)	0.98
Platelet-to-lymphocyte ratio	255 (163-416)	342 (237-438)	0.09
Sodium (mg/dL)	138 (136-140)	138 (136-140)	0.55
White blood cell count (10^3^/uL)	7.4 (5.5-10.4)	7.7 (5.9-11.0)	0.63

Data presented as % (n) or median (IQR).

## Discussion

The growing incidences of cancer and simultaneous advances in cancer treatment have led to a larger number of patients surviving for longer periods and developing bone metastases. The increased proportion of patients with bone metastases has important implications for prognosis and care. Care is primarily focused on pain relief and quality of life. In addition to being painful, bone lesions pose a risk for pathologic fracture. In patients with a longer life expectancy, prophylactic stabilization may be a suitable preventive measure. However, for patients with an estimated survival of less than 3 months, the morbidity, cost, and risk of short-term complications after stabilization must be weighed against that of fracture risk during this shorter time span. Recently, we developed a machine-learning algorithm based on demographics, clinical characteristics, and laboratory values to assess these differences on life expectancy. On geographic validation in the United States, Taiwan, and the Netherlands, the algorithm showed great promise, with AUCs ranging from 0.76 to 0.84 [22, 29]. However, in addition to geographic external validation, as cancer treatment regimens evolve over time, these algorithms should also be temporally validated. In this study, we evaluated the SORG-MLA on a more recent (2016 to 2020) dataset. The discriminatory performance of the model (the extent to which it can distinguish between the different outcomes) was reasonable (0.78 for 90-day survival and 0.76 for 1-year survival). However, the calibration intercept and slope showed values that indicate overestimation of mortality at the 90-day and 1-year timepoints, highlighting the need for ongoing temporal validation of the algorithm. The SORG-MLA is freely available at https://sorg-apps.shinyapps.io/extremitymetssurvival/.

### Limitations

First, the retrospective design and its accompanying restrictions pose a practical limitation to this study. Because of missing data in records, missing data needed to be imputed, and so our analysis was partly based on assumptions rather than on measurements. However, the missForest method for multiple imputation is considered an appropriate approach to handle missing data [15, 28]. Second, our subgroup analysis was limited by a small sample size and the retrospective nature of our data. Because of this, comparison of the various treatment regimens and patients’ response to this treatment could not be closely investigated. Ideally, the response to treatment should have been categorized because there are several gradients of response to cancer treatment. Third, the use of immunotherapy should also have been categorized, because some treatment regimens may have a more profound effect on prognosis. Considering this limitation, readers should not conclude that this finding is the sole reason for the degraded performance of the SORG-MLA. However, we could conclude from our data that the risk of mortality of patients who were treated with novel immunotherapeutic regimens was overestimated often, indicating that the MLA provides additional information. Fourth, it is not always clear whether patients will receive immunotherapy in the future. This poses a practical limitation to these machine-learning models because one can only use data that are available to the clinician at the point of decision-making. Immunotherapy is sometimes used as a last-resort medication, which means that patients have exhausted all other treatment modalities and therefore have greater morbidity than patients who have not yet received chemotherapy or hormonal therapy. Clinicians should consider that, in the end, the treatment effect is the most important for the patients’ prognoses, and it is therefore important that clinicians adjust the model’s prediction based on their own clinical experience. Lastly, the validation cohort originated from two hospitals in an urban area in the Northeastern United States. Our results are therefore more generalizable to academic hospitals in developed countries. To confirm geographic and temporal generalizability, validation should be performed using data from multiple institutions on multiple continents. Ideally, we would integrate these into our electronic medical records and provide a constant feedback loop to evaluate diagnostic accuracy with every patient evaluated by the algorithm. To date, none of the SORG algorithms have been incorporated into electronic medical records because of many challenges, such as institutional review board and legal regulations, ethical considerations, and data and model governance [25]. Because continuous retraining is not yet possible, we recommend frequent (at least every 5 years) validation and retraining of these models. Expected deflections in the outcome, changes in the patient population, or innovations in treatment strategies should be the leading driver for deciding this timeframe.

### Performance of the SORG-MLA on Temporal Validation

We found that the SORG-MLA showed decreased discriminative ability in this temporal cohort consisting of 406 patients. The most important reason for this is that overall mortality has decreased in this cohort from a more recent period (28% to 23% for 90-day mortality and 59% to 51% for 1-year mortality). Because of this change, the data on which the model was trained do not mirror the target population, and a decrease in performance seems logical. Another possible reason for this is that the algorithm overestimated the risk of 90-day and 1-year mortality in 38 patients. In the past decade, the use of checkpoint inhibitors (CDK inhibitors, PD-[L]1 inhibitors, and CTLA4 inhibitors) has increased in cancer treatment and they have been proven to be effective for improving patients’ survival in a wide range of malignant diseases [9, 12]. Surprisingly, the patients in the validation cohort were older than those in the developmental cohort, and generally had more aggressive tumors. The fact that the algorithm still overestimated the risk of death suggests that the survival of patients with metastatic bone disease of the extremities has increased even more than what we observed in the current study. A subgroup analysis of patients who had overestimated predictions of mortality showed that these patients were more often treated with novel immunotherapy and had better responses to this treatment. Unfortunately, the current model does not consider these more modern treatment regimens, which could be why some patients’ risk of mortality was overestimated by the algorithm. Apart from the overestimated patients, the overall mortality of patients in the temporal validation cohort was decreased at both timepoints. This could be because of improvements in diagnostics, medical treatment, and surgical and anesthetic techniques, leading to better prognoses for a wide range of cancer types. Because the primary tumor grouping used to develop the model evaluated in the current study is based on patients treated from 2005 to 2008 [13], an evaluation or even revision of this scoring system is warranted. Furthermore, patients in the validation cohort were less often treated for a pathologic fracture than those in the developmental cohort; this could indicate that diagnostics are indeed improving and it could also have contributed to lower mortality, because patients with a pathologic fracture often have a worse prognosis than prophylactically treated patients [6, 19]. Researchers are encouraged to investigate additional possible causes of these improvements and to investigate whether these novel treatment regimens can have added value to models such as the SORG-MLA. Because of a low sample size, we did not attempt this here.

Additionally, we saw some differences in laboratory values; calcium levels were lower and alkaline phosphatase levels were higher. These differences in bone turnover markers could have led to an overestimated risk of death in these patients. There are several possible reasons for this. In a retrospective study, members of our research group found that higher levels of alkaline phosphatase are associated with lower survival in patients with extremity metastatic disease [23]. The threshold for this lower odds of survival was calculated to be 100 IU/L. However, because this falls within the normal range of alkaline phosphatase in the blood (44 to 147 IU/L), it does not directly mean that it is clinically relevant. The algorithm might have overweighed the importance of this variable. For one of the patients for whom the risk of mortality was overestimated, the high level of alkaline phosphatase was attributed as having a higher impact on the risk of mortality than the presence of brain metastases and tumor origin. However, when the alkaline phosphatase levels are extremely high (Supplemental Fig. 1; http://links.lww.com/CORR/B114), patients might have extensively disseminated metastatic bone disease, which can provide more prognostic value than a small brain lesion that does not cause brain swelling and is therefore of little prognostic value.

### Conclusion

The SORG-MLA showed decreased performance in this temporal cohort from the developmental institution. Additionally, the risk of mortality was overestimated in patients in whom immunotherapy was used compared with patients in whom immunotherapy was not used, where the MLA delivered more accurate estimations of mortality. Clinicians are therefore advised to adjust the model’s predictions based on their own experience, considering that patients who are treated at tertiary care facilities in developed countries will have a better prognosis than is depicted by the SORG-MLA. Importantly, patients treated with immunotherapy are more prone to this overestimation. The results of this study show that systematic validation and retraining of these MLAs is of paramount importance because the predictive performance declines over time as treatment regimens evolve. Patients with otherwise poor chances of survival only a few years ago now show better response to treatment. Future researchers should attempt to investigate the usefulness and possible added value of treatment with novel regimens as a variable, preferably in prospective, multicenter approaches, in which these changes can be documented and implemented in the algorithm in real time. The SORG-MLA is available as a freely accessible internet application that can be found at https://sorg-apps.shinyapps.io/extremitymetssurvival/.

## Supplementary Material
