# Supplementary material for: Does the SORG Machine-learning Algorithm for Extremity Metastases Generalize to a Contemporary Cohort of Patients? Temporal Validation From 2016 to 2020
Source: Clin Orthop Relat Res. 2023 May 25;481(12):2419–30. doi: 10.1097/CORR.0000000000002698 (PMC10642892; doi:10.1097/CORR.0000000000002698)
Supplement: Supplementary file 1 [file abjs-481-2419-s001.docx]

**
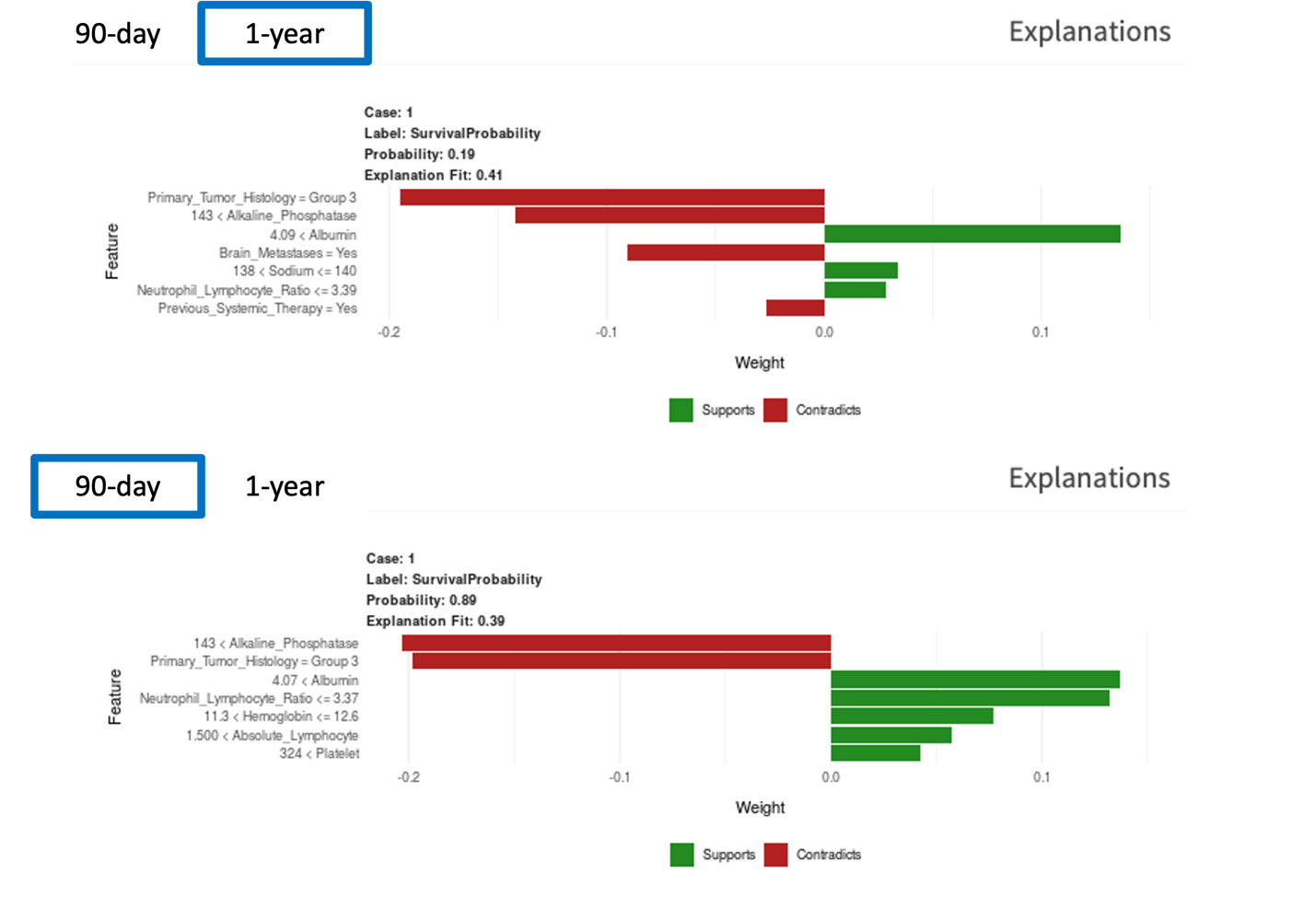
**

**Supplemental Fig. 1** This image shows explanations of survival prediction for one of the patients in the group with overestimated mortality prediction. This 67-year-old man with nonsmall cell lung carcinoma had brain metastases and visceral metastases, and had received prior systemic therapy. His alkaline phosphatase level was 192 IU/L at the time of surgery. In this case, the algorithm attributed a higher risk of mortality to the level of alkaline phosphatase than to the presence of brain metastases and the general tumor characteristics for 1-year survival and 90-day survival, respectively.
